# “Just Because I Don’t Conform to Societal Norms”: A Qualitative Study of Transgender People’s Experiences of Domestic Violence and Coping Methods

**DOI:** 10.7759/cureus.50730

**Published:** 2023-12-18

**Authors:** Pelin Göksel, Ahmet Rifat Şahin, Ömer Böke, Hatice Özyıldız, Gökhan Sarısoy, Aytül Karabekiroğlu, Selcuk Özdin, Ece Turan

**Affiliations:** 1 Psychiatry, Ondokuz Mayıs University, Samsun, TUR; 2 Psychiatry, Faculty of Medicine, Ondokuz Mayıs University, Samsun, TUR; 3 Adult Psychiatry, Ondokuz Mayıs University, Samsun, TUR

**Keywords:** gender incongruence, domestic violence, coping, qualitative research, transgender

## Abstract

Background

Transgender people experience violence in various forms, primarily domestic violence. The aim of this study was to examine transgender people’s experiences of domestic violence and their coping methods.

Materials and methods

This study was conducted using the phenomenological method, one of the five basic qualitative research methods, with 20 transgender participants who applied to Ondokuz Mayıs University, Samsun, Turkey, to start the gender-affirming treatment process. The participants comprised 19 transgender men and 1 transgender woman. A semi-structured interview form was used for data collection. The average interview duration was 75.7 minutes. Audio recordings were used in the interviews, which were then transcribed. The obtained data set was subjected to content analysis.

Results

As a result of the content analysis, three themes emerged: being a transgender individual and the family, experiences of domestic violence, and methods of coping. According to the study results, the participants had experienced domestic violence of different dimensions, primarily psychological violence. Defined gender roles and societal expectations were determined to trigger violent behaviors. The most frequently used coping methods were giving a direct reaction, seeking instrumental-social support, and ignoring the incidents.

Conclusion

Our findings demonstrated that transgender people experience domestic violence at a high rate and that transphobic behaviors are triggered by societal norms. Our results are particularly noteworthy for clinicians regarding the importance of family support and accurate information for transgender people and the coping methods they use most.

## Introduction

The phrase "transgender" is used to describe people whose gender identities or expressions differ from the gender socially attributed to the sex assigned to them at birth. Recent mainstream global medicine no longer classifies transgender and gender diverse (TGD) identities as a mental disorder [1]. The Diagnostic and Statistical Manual Version 5 (DSM-5) includes a diagnosis of gender dysphoria, which focuses on any distress or discomfort accompanying being transgender [2]. In the International Classification of Diseases, Version 11 (ICD-11), gender incongruence is defined as significant incongruence between a person's experienced gender and the assigned sex [3].

Studies focusing on the experiences of transgender people reveal that they face various psychological difficulties such as not accepting their gender identity, worrying about the social consequences of their gender identity, anxiety about the future, fear of rejection, and internalized transphobia. They are exposed to varying degrees of violence in different platforms such as family, school environments, and public areas [4-6]. The form and severity of negative attitudes and behaviors faced by transgender people, due to gender expressions that do not comply with societal normative values, vary according to societal acceptance and understanding, as is the case with every individual in sexual minority status [7-9].

Several researchers have noted that families may reject having a transgender child to prevent stigmatization and loss of social status [10]. A book offering a detailed perspective on honor-based violence stated that in most cultures, the social status of the family is seen as more important than personal welfare. Maintaining an honorable family image is associated with pride, economic opportunities, and positive self-perception [11]. There is thought to be a strong association between family violence experienced by transgender people and concepts of stigmatization and honor [12].

In a survey conducted in the USA in 2017, discrimination experiences of LGBTQ individuals were examined. According to the results of this study, 84% of the transgender participants stated that they believed that there was discrimination against transgender people in the USA, 38% stated that insulting words were used against the group to which they belonged and 28% stated that they had experienced negative and offensive statements against them. In addition, 10% of the transgender participants stated that they experienced discrimination while receiving health care, 22% of them avoided getting health care services with the expectation of discrimination, and 27% of them stated that they were considering moving to another region because of discrimination [13].

The United States Transgender Survey (USTS) examined the negative factors to which transgender people are exposed because of gender differences. It reported that gender identity was not accepted by close family members of 40% of the transgender participants, and 15% were evicted from their homes [14]. A study conducted in Chicago included 78 trans-females and 33 trans-males, and it was reported that 66% of the participants had experienced violence in the home, and these violent behaviors could not be differentiated according to gender or type of violence [15].

No studies have been conducted in Turkey focusing on domestic violence against transgender people. A recent systematic literature review highlighted the scarcity of studies addressing discrimination against LGBTQIs and the lack of official documentation about this issue in Turkey [16]. This review argued that LGBTQIs are systematically subjected to oppression in Turkey, that this oppression results from the patriarchal regime, and that improvements can only be possible through legal regulations.

Coping with stress is defined as the cognitive, emotional, and behavioral actions taken to tolerate difficulties experienced as a result of interaction with the environment, and to reduce the internal and external demands overwhelming one’s own resources [17]. In literature, coping methods are usually categorized as “approach coping” and “avoidance coping”. Approach coping is the use of interventions to cope directly with stressors, such as seeking help, trying to see the positive aspects, planning, and accepting reality, and avoidance coping is the use of interventions such as rejection, substance use, not making an effort, and blaming oneself with the aim of avoiding the stressors and not facing up to emotions [18].

There are few studies that have examined the coping methods of sexual minority groups. A qualitative study examined the coping methods used by transgender people and concluded that the coping methods used were personal, such as gender normative, emotion regulatory, self-affirmative, and cognitive reframing, intrapersonal, such as determining social-relational, preparatory-preventative interventions, and systemic, such as disengagement, resource access, spiritual-religious, and political empowerment [19].

In our study, which aims to examine transgender individuals' experiences of domestic violence and how they cope with these difficulties, we used the qualitative method to understand more deeply complex phenomena such as experiencing and coping with violence. We hope that our study will increase awareness about cases of violence against transgender people and shed light on clinicians in the follow-up of transgender patients on issues such as paying special attention to family interviews and addressing coping methods of them.

## Materials and methods

Study design

This study was designed using the phenomenological method, which is one of the five basic qualitative research methods [20]. As the phenomenological method focuses on the experiences of individuals, it was considered suitable for the evaluation of experiences of transgender people.

Study participants

The sample of the research consisted of 20 participants who applied to Ondokuz Mayıs University Psychiatry Polyclinic between 2018 and 2021 to start the gender-affirming process. Participants were between the ages of 18 and 36. The study participants comprised 19 transgender men and one trans woman. The sample size for the study was determined on the basis of the criteria of “reaching data saturation” [21].

Our clinic is one of the important centers where the gender-affirming process is monitored in Turkey. The number of clients we follow is higher than other centers. Despite this, we only had three trans women clients. We could not reach one of them due to a change of address, and one did not want to participate in the study. For this reason, we were able to include only one trans woman participant in our study. In other studies conducted with individuals applying for the gender-affirming process in Turkey, there is also inequality in terms of gender distribution. It is thought that this situation is due to the less acceptance of feminine behaviors by men in Turkey [22-24].

Preparation of the semi-structured interview form 

The interview questions related to incidents of violence against LGBTQ individuals and the methods of coping with these were prepared with reference to the themes determined in the literature. A pilot interview was conducted. An audio recording was made of the interview, which was then transcribed, and the content was analyzed. After the analysis of the pilot interview, expert opinion was sought and the questions were revised in line with the suggestions.

Some questions in the semi-structured interview form are as follows: Have you disclosed your gender identity to your family? How did they react? How does your family address you? How does this address make you feel? What are your suggestions for a transgender individual at the beginning of the gender-affirming process?

Data collection

All the interviews were conducted face-to-face in the clinic by the first author. The shortest interview lasted 32 minutes, and the longest interview lasted 204 minutes. Ten participants were interviewed once, nine participants were interviewed twice, and one participant was interviewed four times. Audio recordings were taken during the interviews, and the recordings were documented afterward.

Data analysis

Data collected in the interviews were analyzed with content analysis [25]. No computer program was used in the analysis. The data obtained in content analysis were subjected to a systematic coding and categorization process [26]. After repeated reading and interpretation of the transcripts, codes were assigned to the sections that provided semantic integrity. The resulting codes were listed and categorized according to their common points. Then, the categories were grouped under three themes. Coding was done by a single coder. Consolidated Criteria for Reporting Qualitative Studies (COREQ) guidelines were used in the reporting of qualitative data [27]. The data analysis process is exemplified in Table 1.

**Table 1:** Examples of meaning units and codes in the data analysis

Themes	Categories	Codes	Authentic expressions
Being a Transgender Individual and the Family	The Family Factor in the Process of Existence	expectation from family	I wanted to talk to my family. To take my hand and resolve my problem… (p15, 36 years)
Being a Transgender Individual and the Family	Having a Transgender Child	concern	Because I’m known as O’s daughter. O’s daughter has a beard My father is frightened of this. There is a tranny - O’s daughter is a tranny. My father only thinks about what others think, whereas he doesn’t care me… (p14, 24 years)
Experiences of Domestic Violence	Psychological Violence	emotional needs not being met	At home, my mother and I did not look at each other's faces. My mother was acting as if I was dead (p2, 23 years)
Experiences of Domestic Violence	Economic Violence	not giving money	“Don't send him money, let him be broke.” My brother is always trying to punish me with Money (p18, 23 years)
Coping Methods	Approach Coping	researching, informing	You can raise awareness in the family. Examples can be given. They can break out of the mold they are in (p11, 23 years)
Coping Methods	Avoidant Coping	ignoring	I tried not to hear what was said (p6, 19 years )

Validity and reliability

This study used the methods of credibility, transferability, consistency, and assignability recommended by Lincoln and Guba for the evaluation of the validity and reliability of qualitative studies [28]. The interviews had no time restriction, were conducted in the form of a conversation, breaks were given in some interviews or the interaction was continued at the end of the interview. For some participants, there was information exchange about support platforms for gender-related violence and victims of violence, and thus prolonged engagement was achieved. To examine the consistency and assignability, raw data were coded again by the authors. Five participants were sent the findings of the study and were asked to evaluate whether the findings adequately reflected their opinions; member checking was made. Two of the feedback from the participants are as follows: (1) "I think the themes are very appropriate, very well summarized. While reading, there were many places where 'I said yes, definitely'" (p4, 25 years) and (2) "I think the findings reflect reality very well. I hope it will be useful to those who read it" (p3, 20 years).

## Results

The mean age of the study participants was 23.8 years (range, 18-37 years). Only one of the participants was a transgender woman (coded p8). The sociodemographic and clinical characteristics of the study participants are shown in Table 2.

**Table 2:** Sociodemographic and clinical characteristics N/A: Not Available

Characteristics		n (%)
Age (mean, years)		23.8
Gender	Transgender man	19 (95.0%)
	Transgender woman	1 (5.0%)
Education level	Primary school	1 (5.0%)
	High school	7 (35.0%)
	University	12 (60.0%)
Employment status	Employed	9 (45.0%)
	Unemployed	11 (55.0%)
Monthly income	Low	7 (35.0%)
	Moderate	8 (40.0%)
	High	5 (25.0%)
Hormone treatment status	Receiving	13 (65.0%)
	Not receiving	7 (35.0%)
Interventions performed for gender-affirming	Mastectomy	5 (25.0%)
	Hysterectomy	2 (10.0%)
	N/A	15 (75.0%)

The 396 codes derived as a result of content analysis were first collected into 10 categories, and then these categories were discussed under three themes. A detailed explanation of the coding process is presented in Table 3.

**Table 3:** Themes and categories

Themes	Categories	Codes and frequencies
Being a Transgender Individual and the Family	The Family Factor in the Process of Existence	The importance of family support (28)
		Family acceptance (28)
		Get used to the differences over time (14)
		Expectation from family (14)
		An indication of acceptance: desired name (7)
		Adopting male gender more easily (7)
		To think family is right (7)
	Family's view of being a transgender individual	Affectation (6)
		Disease (3)
		Perversion (2)
	Reasons for the Transphobic Attitude of the Family	Social attitudes (9)
		Piety (6)
		Lack of education (4)
	Having a Transgender Child	Concern (29)
		Denial (19)
		Negative expectations (11)
	Family Interventions to Correct (!)	Being forced to change gender expression (12)
		Resorting to religious methods (6)
		Postponing the gender-affirming process (4)
Domestic Violence Experiences	Psychological Violence Experiences	Insult and insulting jokes (25)
		Emotional needs not being met (21)
		Calling by ID name (11)
		Threatening (10)
		Blaming (5)
		Forcing away (4)
		Restricting freedom (2)
		Obstructing healthcare (2)
	Economic Violence Experiences	Not giving money (6)
		Disinheriting (2)
	Physical Violence Experiences	Physical assault (5)
Coping Methods	Approach Coping	Giving a direct reaction (33)
		Seeking instrumental-social support (25)
		Researching, informing (19)
		Being hopeful and real (18)
		Taking a decisive stance (11)
		Being social (5)
	Avoidance Coping	Ignoring (24)
		Moving away from social circle (18)
		Hiding gender identity (18)
		Lying and acting (12)
		Self-harming (11)
		Being transphobic (6)

Being a transgender individual and the family

The Family Factor in the Process of Existence

The participants usually expected their families to accept gender incongruence or to support them in the process they would go through and the challenges they would struggle with, and they attached great importance to this support.

"I wanted to talk to my family. To take my hand and resolve my problem…" (p15, 36 years).

"My father said, 'do whatever you want and behave however you want, and use whatever name you want, it’s not a problem for me. If they do anything bad to you here and you get a reaction, come and tell me'. I felt good because of this, as if I had a huge mountain at my back and nobody can interfere with me. And afterwards my father always supported me greatly…" (p3, 20 years).

Family's View of Being a Transgender Individual

The participants stated that their families believed gender incongruence had occurred because of an affectation or a disease, or that it was a perversion.

"My mother said you are imitating somebody. Who can I be imitating? Am I stupid that I want to have such a difficult, expensive operation? I’m not making this up, I wanted to have the body I was born with and to live in that way, but it’s not possible…" (p2, 23 years).

Reasons for the Transphobic Attitude of the Family

The participants put forward the reasons for their families' transphobic attitudes, particularly societal gender roles, religiousness, and lack of education.

"Although my older brother was aware of who I am, he felt embarrassed about this, and felt he has the right to exhibit these behaviours, saying that I just don’t conform to societal norms…" (p8, 20 years).

Having a Transgender Child

The participants stated that their families were concerned about the family positions and negative things that could happen to their children because of their trans status, and they tended to deny gender incongruence.

"My family hide this from other people, they are ashamed. Mostly my father. He says there’s nothing like this in our family, he’s ashamed by the people. Because I’m known as O’s daughter. O’s daughter has a beard My father is frightened of this. There is a tranny- O’s daughter is a tranny. My father only thinks about what others think, whereas he doesn’t care me care…" (p14, 24 years).

"There was a girl called O, who lived across from us and we used to write letters to each other. My mother saw our letters but she didn’t say anything, she acted as if she hadn’t seen them…" (p15, 36 years).

Family Interventions to Correct (!)

The participants stated that their families had applied some disturbing methods for this purpose.

"My older sister went to a lot of trouble to try to change me. She tried to dress me and do my hair..." (p13, 22 years).

"One day when I went to my grandmother’s house I saw that all the neighbours were gathered there. They filled a large bowl with water, laid a sheet over it, then scattered my underwear and all my clothes on the floor. They made me sit, and next to the water they chanted prayers then told me to get dressed and I would be corrected. I’m embarrassed about that event, with all my underwear on the floor and that it was done with so many people…" (p7, 25 years).

Experiences of domestic violence

Psychological Violence

The participants stated that they were exposed to psychological violence, primarily to verbal violence, and their emotional needs were not met through being threatened, evicted from the home, forced to be at a distance, having limited freedom, and being prevented from accessing healthcare services.

"My mother has given up on me, But she’s attached to my brother he cries, my mother hugs him and cries. I didn’t do anything to be deprived of these…" (p7, 25 years).

"My father says that if I change my gender, be aware that you are dead. I think he can do that" (p14, 24 years).

 "My father made me wear a skirt and had his car cleaned just so that the people in the apartment would recognize me as a girl" (p15, 35 years).

"My older brother is ashamed that I am his brother, calling me gay, faggot, tranny. You want this for yourself, you do it to attract attention, there’s nothing like this in your nature, you are using this, I hate you, I don’t want a brother like you" (p8, 20 years).

"I said I was going to start the process. They said get out of here and do whatever you want" (p19, 19 years).

Economic Violence

Some participants stated that their families refused to give the necessary economic support to their children because of their gender incongruence. Therefore, many participants had to work from a young age.

"My uncle told my father to cut me out of his will. I was in the next room and heard this, but in any case, I don’t have my eyes on any inheritance. I earn my own money" (p19, 19 years).

Physical Violence

Some participants shared their experiences of physical violence.

"I'll never forget one day my brother had me up against the wall and squeezed my throat until I couldn’t breathe" (p8, 20 years).

"My father once beat me with a hosepipe because of the way I am" (p12, 22 years).

Coping methods

Approach Coping

The participants used approach coping methods to deal with domestic violence by primarily giving a direct reaction and seeking instrumental-social support through research and information, taking a decisive stance, being hopeful, being real, and self-acceptance. 

"When I first opened up to my mother she told me to go and live far away. I said no, I’m a grown adult. I’m not going to live how other people say I should - like it or not" (p10, 37 years).

"My mother tried to force me to dress like a girl at weddings but I never did. I would have to pull and drag her to the men’s clothes departments" (p5, 19 years).

"What will happen if I pass the university exam? My father won’t send me any money and again I'll have to work, so logically if I want to be free as soon as possible I have to find work first. I applied for jobs, was called for an interview, then started working and I’ve been working since that day" (p7, 25 years).

"You can make the family more aware. You can give examples, then they can get out of the mould they are in" (p11, 23 years).

Avoidance Coping

The participants used avoidance coping methods to deal with the domestic violence primarily by not reacting to or ignoring it, or distancing themselves from the family, using means that would not hurt themselves such as telling lies and taking on a role.

"If you go down this path you have to mask your feelings. Because people put so much pressure on you. If you don’t ignore what people are saying, it will drive you to suicide. Just look straight ahead and don’t listen to who said this and that" (p20, 20 years).

## Discussion

The aim of this study was to examine how transgender people experience the phenomenon of domestic violence and how they deal with it within a phenomenological framework. In this qualitative study, three themes were identified: being a transgender individual and the family, domestic violence, and coping methods. Throughout the interviews, focus was maintained on the experiences shared by the participants, adherence to their expression style was ensured, and care was taken not to reduce the data to an ordinary study object.

According to the study results, the concept of "family" was seen to be closely related to "support," with great importance attached to this support, and expectations from the family were related to support and acceptance. Previous studies focusing on the families of young transgender people have shown the importance of family support and that it is a protective factor in the struggle with minority stress [29, 30]. In a qualitative study by Budge et al., family support was seen to have an important role in coping with gender incongruences [31].

The participants in the study believed that the pre-formed societal gender roles laid the groundwork for the transphobic behaviors of their families. Additionally, the greatest concern of families related to the gender differences of their children was that the social standing of the family would be damaged. When the literature related to the reasons for transphobic attitudes of the family was examined, it can be seen that it is mostly weighted toward societal gender roles and societal expectations, consistent with the findings of this study [32, 33]. In another study addressing the concerns of the families of children with a gender difference, it was concluded that the families were more worried about negative reactions from family members and people outside the family discussing the situation, rather than the child’s health and interventions for gender conformity [34]. In parallel with the results of the current study, similar studies on the subject have shown that the social status of the family seems to be more important than the welfare of the transgender child [7, 11].

Giordano suggested that many factors such as religion, politics, and education play a role in the expectation of gender roles [35]. However, it should be noted that the relationship between families' religious beliefs and transphobic behaviors is not direct either in our study or in the literature. This situation is thought to be caused by cultural characteristics rather than religious beliefs. Similarly, Morris, in his work on honor-based violence, claimed that honor is not religious, but originated from cultural characteristics, even though it seems to be related to religion [36].

The current study results obtained on the theme of Experiences of Domestic Violence showed that the participants experienced domestic violence mostly in a psychological form, through their emotional needs not being met and through verbal humiliation, taunting, and mockery. Previous studies have also drawn attention to emotional neglect and verbal violence, especially in the nature of emphasizing gender difference [37]. It has been suggested by some researchers that transgender people become a target for abusers as they are thought to be defenseless against verbal attacks about sexuality with a negative meaning [38, 39]. Moreover, it can be seen in the literature, consistent with the current study findings, that transgender people are exposed to physical violence, verbal violence, economic violence, and behaviors such as being forced into changing gender expression, forced into interventions to comply with the birth-assigned gender, being evicted from the home, or being imprisoned at home [5, 9].

When evaluating the coping methods in this study, the data were categorized under the sub-themes of approach coping and avoidance coping. Although the aim was not to compare the frequency of the methods selected, it was concluded that approach coping methods were more often used by the participants. In contrast, reports in the literature state that individuals with a gender difference seem to use avoidance coping methods more often [40]. This difference may be due to the fact that our sample was composed of the group that applied for the gender-affirming process. Despite all the environmental and internal difficulties, starting this process requires using approachable coping methods.

According to the data obtained in the current study, participants tried to deal with the problems they encountered with approach coping methods, using methods such as informing and seeking instrumental and social support, and giving a direct reaction. In a previous qualitative study that examined the coping styles of transgender young adults, the participants stated that direct confrontation was a useful coping method and they recommended that individuals from sexual minority groups use this method when they encounter violent behaviors. Other coping methods recommended in that study were self-acceptance, following your own moral compass, being hopeful about the future, gaining economic independence, and using sources of social support, and these were seen to be similar to the methods used by the current study participants [41]. Some participants dealt with a minority stressful incident by trying to see the bright side and being hopeful. This method has usually been dealt with as the “Cognitive Reframing” method in studies related to transphobic and homophobic violence [42].

In the sub-theme of Avoidance Coping, the conclusion was reached that the participants often ignored gender-related violence, chose to remain unresponsive, and chose isolation for protection. Previous studies have also shown that ignoring problems is a means often used by a sexual minority group to cope with discrimination and violence [43]. Moreover, the current study is consistent with findings in the literature that individuals in a sexual minority group experience a high level of internalized stigmatization, which makes them more vulnerable to violence directed at gender from external sources, and therefore they prefer to hide sexual differences [44].

Study strengths and limitations 

Although there are studies focusing on the violence experiences of the gender minority group in the literature, studies focusing specifically on the domestic violence experiences of transgender people are very limited. Therefore, examining the violent experiences of transgender people by focusing on a specific area is one of the strengths of our study. Qualitative research methods reduce the distance between the researcher and the participant and help examine the problem from the inside. Examining a phenomenon such as the experience of violence, which is difficult to fully grasp with quantitative methods, with a qualitative method based on interviews, is one of the strengths of our study.

The limitations of the study include using only interviews as the data collection method, the lack of data diversity, and that the interviews were held in the polyclinics where the participants were being followed up during the gender-affirming process, with some participants being followed up by the researcher. This may have prevented some participants from being sufficiently open in their responses. Additionally, in our study, data were collected through face-to-face interviews. Data collected through focus groups in our specific sample of transgender participants could increase reliability, which is one of the validity and reliability measurements used in qualitative research.

As this study was conducted in a single center and the sample group was formed of those who had presented for the gender-affirming process, the results cannot be generalized. A further limitation was the imbalance of gender distribution in the sample. The study participants were disproportionately trans-male, as is the case for most research on the gender-affirming process in Turkey, for the reasons stated in the Materials and Methods sections. If we could have included more trans women participants in our study investigating transgender people's experiences of domestic violence, it might have resulted in different results and we might have reached some specific themes. Although there are not many studies focusing on the experiences of domestic violence against trans women, when the literature is examined, there are qualitative studies that strikingly reveal intimate partner violence and sexual violence against trans women. For this reason, the fact that there was only one trans woman participant in our study may not have made the necessary contribution to the literature on trans women's violence experiences [45, 46].

## Conclusions

The main reason for domestic violence against transgender people is the norms and expectations molded by society, in other words, the heteronormative societal organization. If gender differences are seen as a threat to heteronormativity, this leads to transphobia, and violence is legitimized. So, if the presence of a transgender child in the family is perceived as a threat to family status, this gives rise to violence. It was concluded that both approach coping and avoidance coping methods are frequently used, and when the young mean age of this sample is taken into consideration together with the nature of the stress to which they were exposed and that there were usually insufficient resources, it can be seen that their approach coping skills were more developed than expected. This could be due to the study sample having been formed of those who had presented for the gender-affirming process and could have therefore already started this process by overcoming several obstacles with approach coping methods.

Our study has revealed how difficult events transgender people face and the importance of the family factor. Based on these results, it can be suggested that clinicians should pay special attention to the interviews they have with the families of transgender people. In addition, clinicians should realize that transgender people use various coping methods to combat all these difficulties, and adaptive coping methods should be supported. In the future, quantitative studies on the subject and the use of different sampling methods (e.g., snowball sampling method), focusing on different forms of violence (e.g., intimate partner violence) and qualitative studies using different interview questions will be able to make a greater contribution to our understanding of the experiences of violence.
